# p53 and the E3 Ubiquitin Ligase MDM2 in Glaucomatous Lamina Cribrosa Cells

**DOI:** 10.3390/ijms252212173

**Published:** 2024-11-13

**Authors:** Kealan McElhinney, Mustapha Irnaten, Jeffrey O’Callaghan, Colm O’Brien

**Affiliations:** 1 UCD Clinical Research Centre, Mater Misericordiae University Hospital, D07 R2WY Dublin, Ireland; 2 Department of Ophthalmology, Mater Misericordiae University Hospital, D07 R2WY Dublin, Ireland; 3 Ocular Genetics Unit, Smurfit Institute of Genetics, Trinity College, University of Dublin, D02 PN40 Dublin, Ireland

**Keywords:** ophthalmology, glaucoma, p53, mdm2, apoptosis, fibrosis, proliferation

## Abstract

Lamina cribrosa (LC) cells play an integral role in extracellular matrix remodeling and fibrosis in human glaucoma. LC cells bear similarities to myofibroblasts that adopt an apoptotic-resistant, proliferative phenotype, a process linked to dysregulation of tumor suppressor-gene p53 pathways, including ubiquitin-proteasomal degradation via murine-double-minute-2 (MDM2). Here, we investigate p53 and MDM2 in glaucomatous LC cells. Primary human LC cells were isolated from glaucomatous donor eyes (GLC) and age-matched normal controls (NLC) (n = 3 donors/group). LC cells were cultured under standard conditions ± 48-h treatment with p53-MDM2-interaction inhibitor RG-7112. Markers of p53-MDM2, fibrosis, and apoptosis were analyzed by real-time polymerase chain reaction (qRT-PCR), western blotting, and immunofluorescence. Cellular proliferation and viability were assessed using colorimetric methyl-thiazolyl-tetrazolium salt assays (MTS/MTT). In GLC versus NLC cells, protein expression of p53 was significantly decreased (*p* < 0.05), MDM2 was significantly increased, and immunofluorescence showed reduced p53 and increased MDM2 expression in GLC nuclei. RG-7112 treatment significantly increased p53 and significantly decreased MDM2 gene and protein expression. GLC cells had significantly increased protein expression of αSMA, significantly decreased caspase-3 protein expression, and significantly increased proliferation after 96 h. RG-7112 treatment significantly decreased COL1A1 and αSMA, significantly increased BAX and caspase-3 gene expression, and significantly decreased proliferation in GLC cells. MTT-assay showed equivocal cellular viability in NLC/GLC cells with/without RG-7112 treatment. Our data suggests that proliferation and the ubiquitin-proteasomal pathway are dysregulated in GLC cells, with MDM2-led p53 protein degradation negatively impacting its protective role.

## 1. Introduction

Glaucoma is a chronic-progressive optic neuropathy and a leading cause of irreversible blindness worldwide [1], estimated to affect approximately 76 million people in 2020 [2]. The primary site of glaucoma-related damage is the lamina cribrosa (LC) region of the optic nerve head (ONH) [3], a three-dimensional structure composed of perforated elasto-collagenous cribriform plates [4] that provides structural support to retinal ganglion cell (RGC) axons as they exit the eye to form the optic nerve [5].

Experimental studies have shown that intraocular pressure (IOP) elevation will result in a characteristic ONH cupping [6] and a thickened, stiffened, and posteriorly displaced LC [7,8]. Later, LC plates undergo shearing and collapse due to exuberant extracellular matrix (ECM) deposition [4] and subsequent pathological ECM remodeling and fibrosis [9]. This process culminates in a thin, fibrotic, architecturally altered LC [10,11] that obstructs RGC axon axoplasmic flow [12,13] and leads to progressive degeneration of RGC axons and associated irreversible vision loss [14,15].

Previous work by our research group suggests that resident glial fibrillary acid protein (GFAP) negative LC cells play an integral, crucial role in ECM remodeling and fibrosis at the ONH in glaucoma [16]. Significantly, LC cells bear similarities to myofibroblast cells known to be responsible for fibrotic disease development elsewhere in the human body [17,18]. These similarities include constitutive expression of alpha-smooth muscle actin (αSMA), collagen type 1 (COL1A1), elastin, and fibronectin, as well as bone morphogenic proteins (BMPs) [19]. Furthermore, LC cells exposed to cyclic mechanical stretch [20], oxidative stress [21], hypoxia (ONH ischaemia) [22], and TGF-β1 [23] adopt a profibrotic response state that results in upregulated ECM gene expression [24,25].

Recently, key research groups in fibrosis have shown that myofibroblasts responsible for systemic fibrotic disease development adopt an apoptotic-resistant, highly proliferative phenotype to perpetuate fibrosis [26]. Significantly, this pathological adaptation has been linked to dysregulated expression of tumor suppressor gene p53 [27]. In the case of LC fibrosis, this is substantiated by previous similar experiments by our research group in 2015 showing reduced expression of cell cycle inhibitors p27 and p57 with associated increased proliferation in glaucomatous LC cells [28]. Recently, in a similar fashion, Tenon’s ocular fibroblasts from glaucomatous eyes were shown to demonstrate increased expression of fibrosis, ECM remodeling, inflammation, and apoptosis [29].

p53 is a potent transcription factor [29] that is activated in response to diverse stresses and environmental insults and is responsible for the induction of cell cycle arrest, apoptosis, or senescence [30]. p53 therefore prevents the emergence of transformed cells with genetic instabilities, preventing cancer onset and development [31], earning its title as the “Guardian of the Genome” [32]. In most, if not all, human cancers, inactivation of p53 disrupts its ability to suppress carcinogenesis, thus transforming the “Guardian of the Genome” into a “Rebel Angel” [33]. In a similar fashion, p53 inactivation has recently been linked to fibrotic disease development [27].

One mechanism of p53 inactivation is negative regulation by the ubiquitin-proteasome system via E3 ubiquitin ligase murine-double-minute 2 homolog (MDM2) [34,35]. MDM2 and p53 participate in an autoregulatory feedback loop [29], whereby p53 positively regulates MDM2 expression by binding to its promoter region, and the resultant increased MDM2 expression leads to MDM2 negatively regulating and inactivating p53 [36]. To facilitate this negative regulation, MDM2 binds and directly blocks the N-terminal transactivation domain of p53, inhibiting p53s transcriptional activity [37]. MDM2 also prevents p53 from interacting with transcriptional co-activators and recruits transcriptional co-repressors to p53 [38]. Furthermore, MDM2 promotes p53 exportation out of the nucleus to the cytoplasm, where MDM2 targets p53 for ubiquitin-dependent proteasomal degradation [39]. MDM2 serves as an E3 ubiquitin ligase via its C-terminal RING finger domain and ubiquitinates p53 at several lysine residues [40].

A greater understanding of the p53-MDM2 interaction has enabled the emergence of novel therapeutics that aim to inhibit MDM2 binding to p53 and thus ensure p53 stabilization and activation [41]. These synthetic compounds work by preventing the transactivation domain of p53 binding to a deep hydrophobic pocket on MDM2, specifically targeting three amino acid residues (Phe19, Trp23, and Leu26) in p53 that are primarily responsible for this protein-protein interaction [42]. Amongst the earliest investigated were the imidazoline derivatives (better known as nutlins), especially nutlin 3a [43]. Nutlin 3a mimics the aforementioned three critical residues in p53 necessary for MDM2 interaction, acting as a competitive inhibitor of p53 binding to MDM2 [34]. Preclinical studies showed that nutlin 3a increased p53 concentrations, enhanced apoptosis, and decreased tumorigenicity in p53 cancer cells [42].

Our research aimed to categorize the role of p53, MDM2, and the ubiquitin-proteasomal system in glaucomatous LC cells and explore how it interacts with glaucomatous LC cell fibrosis, proliferation, and apoptosis. Additionally, we aimed to elucidate the effect that p53-MDM2 interaction inhibitor (nutlin RG-7112) treatment may have on cellular proliferation and apoptosis in glaucomatous LC cells. A greater comprehension of the mechanisms driving fibrotic glaucomatous ONH remodeling is of the utmost importance for the development of novel therapeutic strategies.

## 2. Results

### 2.1. Evaluating Baseline p53 and MDM2 Expression

**Figure 1:**
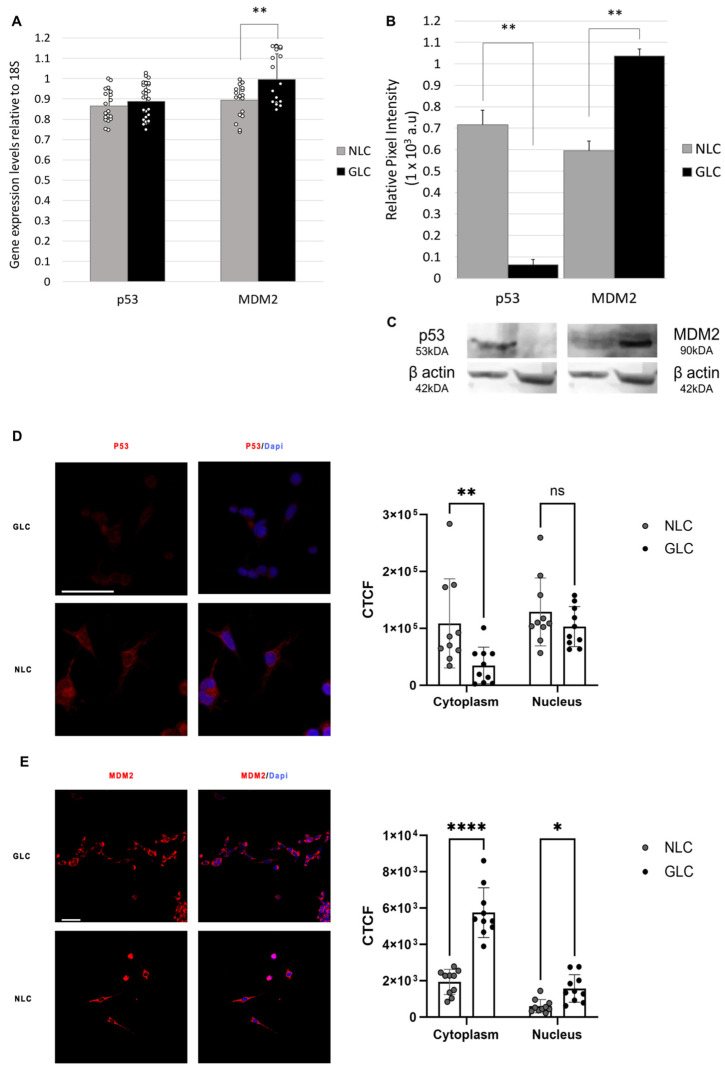
(**A**) RT-qPCR analysis of p53-MDM2-Ubiquitin pathway gene expression in NLC and GLC cell groups (n = 3 donors/group) (technical replicates N = 3) (Unpaired *t*-test, ** *p* < 0.01) (SD error bars). (**B**) Graphical representation of western blot analysis of average protein expression levels of p53 and MDM2 in NLC and GLC cell groups (n = 3 donors per group) (technical replicates N = 3) (Unpaired *t*-test, ** *p* < 0.01) (SD error bars). (**C**) Representative western blot showing p53 and MDM2 protein expression levels in NLC and GLC cell groups (n = 3 donors/group) (technical replicates N = 3). (**D**,**E**) Immunofluorescence microscopy subcellular localization of p53 (**D**) and MDM2 (**E**) NLC and GLC cells (n = 3 donors per group) (technical replicates N = 3) with corresponding corrected total cell fluorescence (CTCF) quantification (Two-way ANOVA, * *p* < 0.05, ** *p* < 0.01, **** *p* < 0.0001, ns means not not significant) (SD error bars). Scale bars represent 50 µm.

To assess p53 and MDM2 differential expression in NLC and GLC cells, we first examined their gene transcription profiles using RT-qPCR (n = 3 donors per group). Results showed that p53 had equivocal gene expression in NLC cell (0.87 ± 0.08) and GLC cell (0.89 ± 0.08) groups (*p* = 0.458), while MDM2 had significantly increased gene expression in GLC cells (1.00 ± 0.13) compared to NLC cells (0.89 ± 0.08) (*p* = 0.005) (Figure 1).

Next, we evaluated protein expression levels of p53 and MDM2 in NLC and GLC cells Figure 1C shows a representative western blot for p53 and MDM2 (n = 3 donors per group). Western blotting analysis of the average protein expression of p53 showed a significant decrease in protein expression in GLC cell (0.06 ± 0.03) versus NLC cell (0.72 ± 0.07) groups (*p* < 0.001) (n = 3 donors per group). For MDM2, average protein expression determined by western blot analysis revealed MDM2 protein expression levels were significantly elevated in GLC cell (1.04 ± 0.03) versus NLC cell (0.59 ± 0.05) groups (*p* < 0.001) (n = 3 donors per group).

Immunofluorescence microscopy was performed on NLC and GLC cell groups to determine the cellular localization of genes of p53 and MDM2. Results showed equivocal p53 fluorescence staining in both the cytoplasm and the nucleus of NLC cells. In comparison, GLC cells showed a significant decrease in p53 staining in both the cytoplasm and nucleus (Figure 1D). Immunofluorescence microscopy also demonstrated that MDM2 staining was mainly located in the cytoplasm in NLC cells. In GLC cells, MDM2 fluorescence signal levels were significantly increased in both the nucleus and cytoplasm in GLC cells (Figure 1E).

### 2.2. p53-MDM2 Interaction Inhibitor RG-7112 Treatment Effect on Gene Expression and Protein Expression of p53 and MDM2

Next, we treated NLC and GLC cells (n = 3 donors per group) with p53-MDM2 interaction inhibitor RG-7112 (ab235500, Abcam, Cambridge, UK) to determine the effect on the p53-MDM2 ubiquitin pathway gene and protein expression levels.

**Figure 2:**
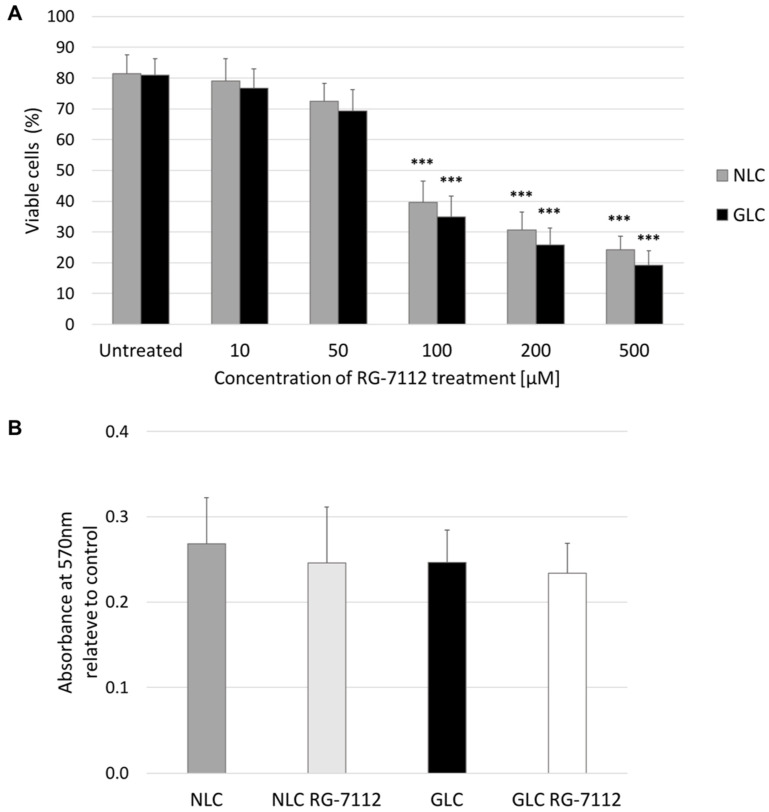
(**A**) Trypan blue exclusion test of cell viability demonstrating the percentage of viable NLC and GLC cells (n = 3 donors per group) (technical replicates N = 3) versus concentration of RG-7112 used. The graph shows a significant dose-dependent cytotoxic effect on NLC and GLC cells with a RG-7112 concentration of 100 μM and above compared with untreated controls (Paired *t*-test, *** *p* < 0.001) (SD error bars). (**B**) MTT cell viability assay reading at 4 h demonstrating the absorbance of 570 nm relative to control for NLC and GLC cells at baseline and with and without 10 μM RG-7112 treatment. The graph shows equivocal cellular survival rates between NLC and GLC groups (n = 3 donors per group) (technical replicates N = 3) with no related cytotoxic effect of 10 μM RG-7112 on either NLC or GLC (n = 3 donors per group) (Unpaired *t*-test, *p* > 0.05) (SD error bars).

Firstly, to elucidate the cytotoxic effect of RG-7112, we treated NLC and GLC cells with varying concentrations of RG-7112 (untreated, 10 μM, 50 μM, 100 μM, 200 μM, 500 μM). The percentage of viable cells (%) was recorded through the use of a trypan blue exclusion test of cell viability [44]. We determined that an acceptable percentage of viable cells after treatment to be greater or equal to 70%. As shown in Figure 2A, RG-7112 treatment resulted in loss of NLC and GLC cell viability in a dose-dependent manner. Results show that there was no significant change in the percentage of viable normal (81.5 ± 6.1) and glaucoma (81.0 ± 5.2) LC cells without RG-7112 treatment (*p* = 0.930). Furthermore, the percentage of viable cells in NLC and GLC cells treated with RG-7112 concentrations of 10 μM (NLC 79.0 ± 7.2) (*p* = 0.681) (GLC 76.6 ± 6.3) (*p* = 0.408) and 50 μM (NLC 72.5 ± 5.7) (*p* = 0.138) (GLC 69.4 ± 6.9) (*p* = 0.080) were not significantly different compared to untreated controls, and therefore it was deemed there was no cytotoxic (apoptotic) effect of RG-7112 at these concentrations.

Higher concentrations of RG-7112 were found to have a dose-dependent cytotoxic effect on human LC cells. Results show that there was a significant reduction in the percentage of viable NLC and GLC cells (%) compared to their untreated controls at RG-7112 concentrations of 100 μM (NLC 39.6 ± 6.9) (*p* = 0.001) (GLC 34.9 ± 6.7) (*p* = 0.001), 200 μM (NLC 30.6 ± 5.8) (*p* < 0.001) (GLC 25.8 ± 5.5) (*p* < 0.001), and 500 μM (NLC 24.3 ± 4.4) (*p* < 0.001) (GLC 19.2 ± 4.7) (*p* < 0.001). Additionally, observation of the cellular behavior of NLC and GLC cells that received RG-7112 treatment found that LC cells kept their characteristic cellular morphology with up to, and including, 50 μM RG-7112 treatment.

Based on the results and literature review [19,27,45,46], we decided to treat NLC and GLC cells with 10 μM (μmol/L) RG-7112. NLC and GLC cells were maintained in standard cell-culture conditions for 48 h, then serum-free media for 12 h, followed by 48 h of cell culture in either untreated serum-rich media or 10 μM RG-7112 groups (n = 3 donors per group).

MTT cellular viability assay demonstrated that NLC and GLC cells had equivocal cellular survival rates (*p* = 0.345) with absorbance readings of 0.27 ± 0.05 and 0.25 ± 0.04 respectively (Figure 2B). Furthermore, the MTT assay showed that 10 μM p53-MDM2 inhibitor RG-7112 treatment did not have a significant effect on cellular viability with absorbance readings of 0.25 ± 0.07 for NLC cells (*p* = 0.444) and 0.23 ± 0.04 for GLC cells (*p* = 0.471).

**Figure 3:**
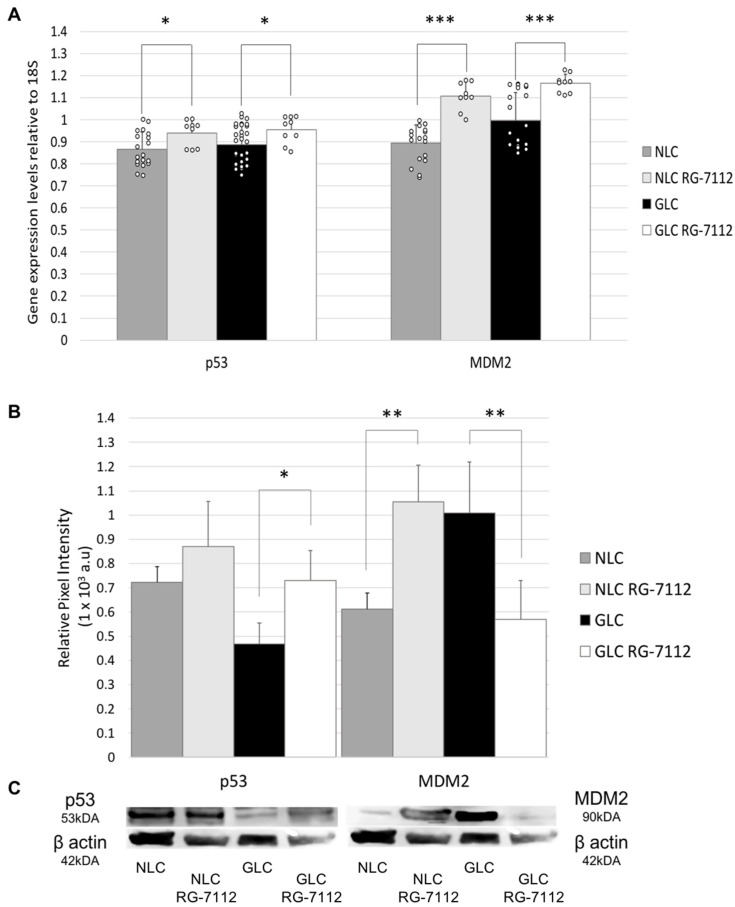
(**A**) RT-qPCR analysis of p53 and MDM2 gene expression in NLC and GLC with and without RG-7112 treatment (n = 3 donors per group) (technical replicates, N = 3) (Two-way ANOVA, * *p* < 0.05, *** *p* < 0.001) (SD error bars). (**B**) Graphical representation of western blot analysis of average protein expression levels of p53 and MDM2 in NLC and GLC cell groups with and without RG-7112 treatment (n = 3 donors per group) (technical replicates N = 3) (Two-way ANOVA, * *p* < 0.05, ** *p* < 0.01) (SD error bars). (**C**) Representative western blot showing p53 and MDM2 protein expression levels in NLC and GLC cells with and without RG-7112 treatment.

When we evaluated the effect of RG-7112 treatment on the transcription profile of NLC and GLC cells (n = 3 donors per group), we found that RG-7112 treatment significantly increased p53 gene expression in treated GLC cells (0.95 ± 0.06) versus non-treated GLC cells (0.89 ± 0.08) (*p* = 0.011). This effect was mirrored in NLC cells, with p53 gene expression significantly increasing in RG-7112-treated NLC cells (0.94 ± 0.06) compared to non-treated NLC cells (0.87 ± 0.08) (*p* = 0.029). Similarly, we also found that RG-7112 treatment significantly increased MDM2 gene expression in treated GLC cells (1.17 ± 0.04) compared with non-treated GLC cells (1.00 ± 0.13) (*p* < 0.001) and treated NLC cells (1.11 ± 0.07) compared to non-treated NLC cells (0.89 ± 0.08) (*p* < 0.001) (Figure 3).

Interestingly, western blot analysis showed that RG-7112 treatment significantly increased protein expression levels of p53 in treated GLC cells (0.73 ± 0.12) compared to non-treated GLC cells (0.47 ± 0.09) (*p* = 0.040) (n = 3 donors per group). In NLC cells, p53 protein expression levels in RG-7112-treated NLC cells (0.87 ± 0.19) and non-treated NLC cells (0.72 ± 0.11) were not significantly different (*p* = 0.303) (Figure 3).

Western blot analysis also showed that MDM2 protein expression levels were significantly decreased in treated GLC (0.57 ± 0.16) compared to untreated GLC (1.00 ± 0.21) (*p* = 0.045) (n = 3 donors per group). This treatment effect was not seen in normal LC cells, with RG-7112-treated NLC (1.06 ± 0.15) showing significantly increased MDM2 protein expression levels compared to untreated NLC (0.61 ± 0.12) (*p* = 0.016) (n = 3 donors per group) (Figure 3).

### 2.3. Expression Analysis of Fibrotic ECM Markers

**Figure 4:**
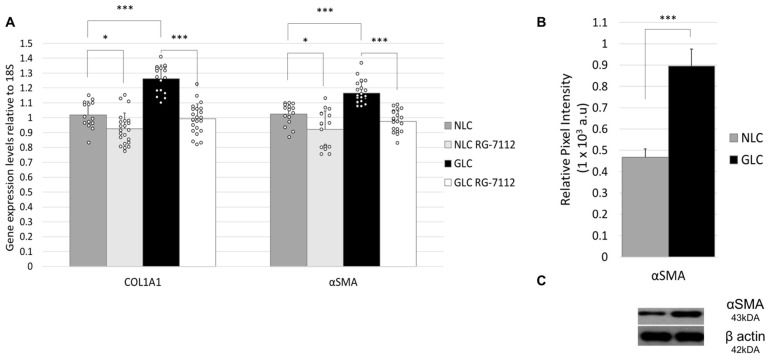
(**A**) RT-qPCR analysis of fibrosis markers COL1A1 and αSMA gene expression in NLC and GLC cell groups with and without RG-7112 treatment (n = 3 donors/group) (technical replicates N = 3) (Two-way ANOVA, * *p* < 0.05, *** *p* < 0.001) (SD error bars). (**B**) Graphical representation of western blot analysis of average protein expression levels of αSMA in NLC and GLC cell groups (n = 3 donors per group) (technical replicates N = 3) (Unpaired *t*-test, *** *p* < 0.001) (SD error bars). (**C**) Representative western blot showing αSMA protein expression levels in NLC and GLC cells.

To assess the expression of markers of fibrotic signaling pathways in NLC and GLC cells, we examined their gene transcription profiles using RT-qPCR (n = 3 donors per group). Results show that gene expression levels of COL1A1 were found to be significantly increased in GLC cells (1.26 ± 0.09) compared to NLC cells (1.02 ± 0.09) (*p* < 0.001). Additionally, αSMA gene expression levels were also found to be significantly increased in GLC cells (1.16 ± 0.08) compared to NLC cells (1.02 ± 0.07) (*p* < 0.001) (Figure 4).

Western blot was used to evaluate differential protein expression levels of markers of fibrosis (αSMA) in NLC and GLC cells. Figure 4 shows a representative western blot for αSMA (n = 3 donors per group). Western blotting analysis showed a significant increase in αSMA protein expression levels in GLC cells (0.89 ± 0.08) versus NLC cells (0.47 ± 0.05) (*p* < 0.001) (Figure 4).

Next, we investigated the effect of RG-7112 treatment on fibrotic ECM genes αSMA and COL1A1. As shown in Figure 4, results show that gene expression levels of COL1A1 were found to be significantly increased in GLC cells (1.26 ± 0.09) compared to NLC cells (1.02 ± 0.09) (*p* < 0.001). RG-7112 treatment resulted in significantly decreased COL1A1 gene expression levels in treated GLC cells (0.99 ± 0.10) (*p* < 0.001) and in treated NLC cells (0.93 ± 0.11) (*p* = 0.010). αSMA gene expression levels were found to be significantly increased in GLC cells (1.16 ± 0.08) compared to NLC cells (1.02 ± 0.07) (*p* < 0.001), with RG-7112 treatment resulting in significantly decreased αSMA gene expression levels in treated GLC cells (0.97 ± 0.07) (*p* < 0.001) and in treated NLC cells (0.92 ± 0.12) (*p* = 0.001).

### 2.4. Expression Analysis of Apoptotic Markers

**Figure 5:**
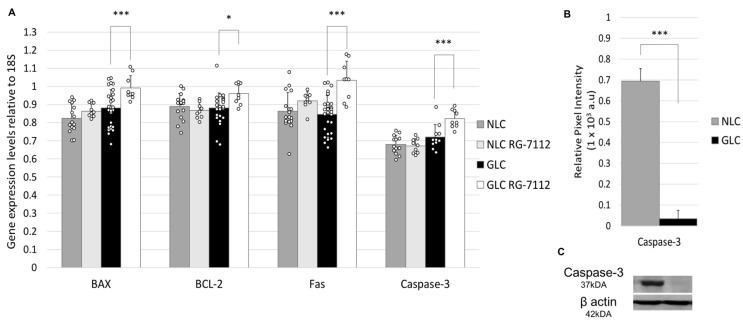
(**A**) RT-qPCR analysis of apoptosis pathway gene expression in NLC and GLC cell groups with and without RG-7112 treatment (n = 3 donors/group) (technical replicates N = 3) (Two-way ANOVA, * *p* < 0.05, *** *p* < 0.001) (SD error bars). (**B**) Graphical representation of western blot analysis of average protein expression levels of caspase-3 in NLC and GLC cell groups (n = 3 donors per group) (technical replicates N = 3) (Unpaired *t*-test, *** *p* < 0.001) (SD error bars). (**C**) Representative western blot showing caspase-3 protein expression levels in NLC and GLC cells.

In a similar fashion, to assess the expression of markers of apoptotic signaling pathways in NLC and GLC cells, we examined their gene transcription profiles using RT-qPCR (n = 3 donors per group). Results show that gene expression levels of apoptotic signaling pathway markers were noted to have equivocal gene expression in GLC cell and NLC cell groups. These included gene expression levels of BAX (GLC cells (0.88 ± 0.10) vs. NLC cells (0.82 ± 0.08) (*p* = 0.059)), BCL-2 (GLC cells (0.88 ± 0.08) vs. NLC cells (0.89 ± 0.07) (*p* = 0.691)), Fas (GLC cells (0.85 ± 0.10) vs. NLC cells (0.86 ± 0.10) (*p* = 0.599)), and Caspase-3 (GLC cells (0.72 ± 0.07) vs. NLC cells (0.68 ± 0.05) (*p* = 0.110)) (Figure 5).

Figure 5 shows a representative western blot for caspase-3 (n = 3 donors per group). Western blotting analysis showed a significant decrease in caspase-3 protein expression levels in GLC cell (0.04 ± 0.03) versus NLC cell (0.69 ± 0.06) groups (*p* < 0.001) (Figure 5).

RG-7112 treatment had a varied effect on apoptotic signaling in NLC and GLC cells. As shown in Figure 5, gene expression levels of BAX were found to be equivocal in GLC cells (0.88 ± 0.10) compared to NLC cells (0.82 ± 0.08) (*p* = 0.059). Following RG-7112 treatment, BAX gene expression levels were significantly increased in treated GLC cells (0.99 ± 0.07) (*p* = 0.004) but did not significantly change in treated NLC cells (0.86 ± 0.04) (*p* = 0.134). When we evaluated BCL-2 gene expression levels, we found they were equivocally expressed in GLC cells (0.88 ± 0.08) versus NLC cells (0.89 ± 0.07) (*p* = 0.691). It was noted that RG-7112 treatment significantly increased BCL-2 gene expression levels in treated GLC cells (0.96 ± 0.06) (*p* = 0.010) while having no significant effect on BCL-2 gene expression levels in treated NLC cells (0.87 ± 0.05) (*p* = 0.475). Fas, a marker of the extrinsic signaling pathway in apoptosis, was found to have equivocal gene expression levels in GLC cells (0.85 ± 0.10) and NLC cells (0.86 ± 0.10) (*p* = 0.599). Fas gene expression levels were significantly increased in RG-7112-treated GLC cells (1.03 ± 0.11) (*p* < 0.001) but Fas gene expression levels did not significantly change in RG-7112-treated NLC cells (0.92 ± 0.05) (*p* = 0.152). Gene expression levels of caspase-3 were found to be equivocal in GLC cells (0.72 ± 0.07) compared to NLC cells (0.68 ± 0.05) (*p* = 0.110). Furthermore, RG-7112 treatment significantly increased caspase-3 gene expression levels in treated GLC cells (0.82 ± 0.05) (*p* = 0.002) but no significant effect on caspase-3 gene expression levels in treated NLC cells (0.67 ± 0.04) (*p* = 0.730) (Figure 5).

### 2.5. Cellular Proliferation

p53 plays an essential role in cell-cycle arrest and the initiation of apoptosis. Given the known proliferative and apoptotic-resistant characteristics of fibrotic myofibroblasts, we examined the proliferative pro-life of human LC cells (untreated and RG-7112-treated).

**Figure 6:**
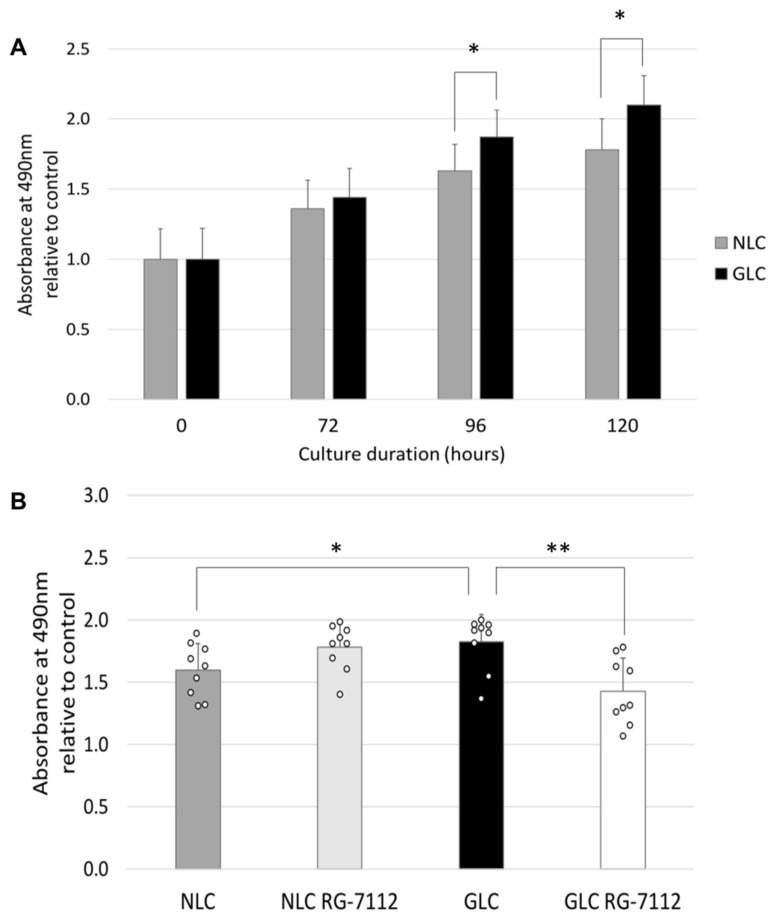
(**A**) MTS cellular proliferation assay readings at 0, 72, 96, and 120 h demonstrate the absorbance of 490 nm relative to control for NLC and GLC cells (n = 3 donors per group) (technical replicates N = 3). Cellular proliferation rates were similar in NLC and GLC cell groups at 72 h but significantly increased in GLC cells at 96 and 120 h compared to NLC cells (Paired *t*-test, * *p* < 0.05) (SD error bars). (**B**) MTS cellular proliferation assay readings demonstrating the absorbance of 490 nm relative to control for NLC and GLC cells with or without 10 μM RG-7112 treatment for 48 h (n = 3 donors per group) (technical replicates N = 3). Cellular incubation for 96 total hours showed a significant increased cellular proliferation in GLC cells versus NLC cells; 48 h of RG-7112 treatment significantly reversed this proliferative profile in treated GLC cells (Two way ANOVA, * *p* < 0.05, ** *p* < 0.01) (SD error bars).

To evaluate cellular proliferation rates, NLC and GLC cells were maintained in standard cell-culture conditions and assessed at 0, 72, 96, and 120 h. MTS assay showed that cellular proliferation rates were similar at 72 h in GLC cells (1.44 ± 0.21) compared to NLC cells (1.36 ± 0.20) (*p* = 0.093) but were significantly increased in GLC cells compared to NLC cells at 96 h (1.87 ± 0.20 vs. 1.63 ± 0.19) (*p* = 0.018) and 120 h (2.10 ± 0.21 vs. 1.78 ± 0.22) (*p* = 0.001) (Figure 6A).

To determine the RG-7112 treatment effect on cellular proliferation rates, NLC and GLC cells were maintained in standard cell-culture conditions for 48 h, then serum-free media for 12 h, followed by 48 h of cell culture in either untreated serum-rich media or 10 μM RG-7112 groups (n = 3 donors per group).

Cellular proliferation: the MTS assay demonstrated that GLC cells (1.83 ± 0.22) exhibited significantly increased cellular proliferation rates compared to NLC controls (1.60 ± 0.22) after 96 h of total incubation (*p* = 0.041) (Figure 6B). Furthermore, 48 h of 10 μM RG-7112 treatment significantly decreased cellular proliferation in GLC cells (1.43 ± 0.26) compared to untreated GLC cells (*p* = 0.003) while having no significant effect on treated NLC cells (1.78 ± 0.19) compared to untreated NLC cells (*p* = 0.069) (Figure 6B).

## 3. Discussion

In this study, we demonstrate that in glaucomatous LC cells, the capability of p53 to regulate cell cycle arrest, apoptosis, and proliferation is significantly affected through dysregulation of MDM2-mediated p53 degradation by the ubiquitin-proteasomal system. p53 is a potent transcription factor [29] responsible for the induction of cell-cycle arrest, apoptosis, or senescence in response to diverse stresses and environmental insults [30]. Subsequently, inactivation of p53 through deletion or mutation disrupts its ability to suppress carcinogenesis [33]. p53 is the most frequently mutated gene in more than 50% of all human cancers [47], with high prevalence in cancers of the breast, prostate, and melanomas [39]. In a similar fashion, p53 inactivation has been linked to fibrotic disease development in multiple organs, including the lung [48], liver [49], and skin fibrosis [50]. Targeting p53 inactivation in cancer and/or fibrosis has focused on negative regulators such as the oncoprotein MDM2 [34,35]. In most situations, MDM2 overexpression is oncogenic and is associated with therapy resistance and poor prognosis [51]. Recent studies have demonstrated that p53-inhibitors MDM2 and MDMX are highly expressed in organ fibrosis and negatively regulate and suppress p53 expression [52,53].

To assess the p53-MDM2-ubiquitin-proteasomal system in NLC and GLC cells, we first investigated the differential expressive profiles of p53 and MDM2. In our study, we demonstrate that there are equivocal p53 gene expression levels between NLC and GLC cells, with a concurrent significant reduction in p53 protein expression levels in GLC cells compared with NLC cells. We also found that overall whole-cell p53 expression was reduced on immunofluorescence in GLC cells compared to NLC cells, with marked reduction noted in both the cytoplasm and the nucleus (Figure 1D). Immunofluorescence analysis of MDM2 expression showed overall whole-cell MDM2 expression to be increased in GLC cells compared to NLC cells, with marked elevation noted in both the cytoplasm and the nucleus (Figure 1E). Our study also demonstrates significantly increased MDM2 gene and protein expression levels in GLC cells compared to NLC cells. These novel findings highlight the dysregulation of the p53-MDM2-ubiquitin proteasomal system at the glaucomatous ONH.

Targeted therapeutics aim to inhibit MDM2 binding to p53 to ensure p53 stabilization and activation [41,42]. Nutlin 3a (an imidazoline derivative)-derived small molecule MDM2 inhibitors phase I clinical trials have shown administration of RG-7112 results in activation of p53, p21, and induction of apoptosis in human tumors [43,54]. Nutlins have also been trialed with in vivo experimental studies in pulmonary [27,52], liver [49], cardiac [55], and renal fibrotic models [56], with resultant restoration of p53 expression and amelioration of fibrosis.

In our study, we initially determined the RG-7112 treatment concentration that would have a treatment effect without cytotoxic side effects on human LC cells. The literature review showed similar in vitro studies on fibroblasts using a concentration of 10 μM (μmol/L) RG-7112 for 48 h [19,45,46]. Analysis of dose-dependent RG-7112 treatment concentrations using the trypan blue exclusion test of cellular viability showed that RG-7112 concentrations of 10 and 50 μM were not cytotoxic as the percentage of viable NLC and GLC cells (%) met an acceptable level. RG-7112 concentrations of 100, 200, and 500 μM were cytotoxic with a significant reduction in the number of viable NLC and GLC cells (%). This study also demonstrated through the MTT cellular viability assay that non-treated NLC and GLC cells had equivocal rates of cellular viability after 48 h. 10 μM RG-7112 treatment for 48 h did not have a cytotoxic effect, as there was no significant change in cellular viability noted in treated versus non-treated groups.

Our study demonstrates the effect that RG-7112 treatment has on the expressive profile of NLC and GLC cells. We found that in vitro RG-7112 treatment had a substantial effect on the rates of p53 degradation, as p53 gene and protein expression levels were significantly increased in treated GLC cells. RG-7112 treatment simultaneously significantly increased MDM2 gene expression levels and significantly decreased MDM2 protein expression levels in treated GLC cells. We hypothesize that glaucomatous LC cells display marked dysregulation of the p53-MDM2-ubiquitin-proteasomal system, resulting in detrimentally low levels of p53 due to aberrant MDM2-mediated degradation. Reduced expression of p53 develops cellular environments devoid of p53s cellular protective responsibilities, leading to uncontrolled cell-cycle propagation and a sustained, pathological fibrotic wound response. The ability of RG-7112 treatment to concurrently significantly increase p53 protein expression and significantly decrease MDM2 protein expression levels shows promise in targeting dysregulation of the p53-MDM2-ubiquitin-proteasomal system at the lamina cribrosa in the pathogenesis of glaucoma.

Following the completion of physiological wound repair, myofibroblasts are terminated through apoptosis [57,58]. Apoptosis is a complex, tightly regulated physiological process that results in programmed cell death [59,60]. However, in chronic/repetitive injury fibrosis, there is a characteristic absence of myofibroblast apoptosis [61,62]. This results in the persistence of activated myofibroblasts that perpetuate the pathological fibrotic disease process [63] and play a prominent role in excessive ECM synthesis, deposition, and remodeling [64,65].

In our study, we also aimed to determine if p53-MDM2-ubiquitin-proteasomal system dysregulation leads to changes in the functional profile of human LC cells. First, we evaluated the baseline expressive profiles of markers of apoptosis and fibrosis in NLC and GLC cells. Results showed that key markers of apoptotic signaling pathway (BAX, BCL-2, Fas, caspase-3) had equivocal gene transcription expression and significantly decreased protein expression (caspase-3) in GLC cells versus NLC cells. Similar analysis of fibrotic markers showed αSMA had significantly increased gene transcription expression and protein expression levels in GLC versus NLC cell groups. Together, these results suggest glaucomatous LC cells are highly fibrotic and that dysfunctional apoptotic signaling confirms the adoption of an apoptotic-resistant, proliferative phenotype.

Next, we evaluated the effect of RG-7112 treatment on apoptotic mechanisms in NLC and GLC cells. We found that RG-7112 treatment significantly increased gene expression levels of markers of the apoptotic signaling pathway BAX, BCL-2, Fas, and caspase-3 in treated GLC cells compared with non-treated GLC cells. This effect was not mirrored in treated NLC cells compared with non-treated NLC cells. RG-7112 treatment also altered the fibrotic expressive profile, with gene expression levels of COL1A1 and αSMA found to be significantly increased in GLC versus NLC cells, with RG-7112 treatment significantly reducing gene expression levels in both groups. Previous experimental studies from our research group have described the fibrotic nature of glaucomatous LC cells in a similar vein to systemic organ fibrosis [66,67,68]. However, this study is the first time it is demonstrated that targeted in vitro therapy of the p53-MDM2-ubiquitin-proteasomal system can reverse pathological mechanisms in fibrotic and apoptotic signaling in glaucomatous LC cells. Similar results have been noted with in vitro RG-7112 treatment of fibrotic lung fibroblasts [27].

As a further means of assessing functional cellular response to RG-7112 treatment, we analyzed cellular proliferation rates using an MTS cellular proliferation assay. At baseline, our study demonstrated that after 96 h of incubation under standard cell-culture conditions, GLC cells exhibit a significantly increased proliferation rate compared to NLC cells; this finding has been consistently demonstrated by our research group [68,69]. When treated for 48 h with RG-7112, we noted a significant decrease in cellular proliferation rates in treated GLC cells compared with non-treated GLC cells. On the contrary, RG-7112 treatment had no significant effect on cellular proliferation rates in treated NLC cells compared to untreated NLC cells. This study again demonstrated that GLC cells adopt a significant proliferative state compared to NLC cells [68,69], which may play an essential role in fibrosis development at the glaucomatous ONH. This study describes targeting and reversing glaucoma LC cell proliferation through p53-MDM2 interaction inhibitor RG-7112 treatment.

Our study demonstrates that glaucomatous LC cells exhibit significant dysregulation of the p53 expressive profile through aberrant MDM2-mediated degradation by the ubiquitin proteasomal system. We hypothesize that reduced expression of p53 in glaucomatous LC cells results in the loss of p53s key protective role in cell-cycle regulation, leading to uncontrolled cell-cycle propagation, elevated cellular proliferation rates, cellular resistance to apoptosis initiation, and perpetuation of a sustained, pathological fibrotic wound response at the lamina cribrosa. In a similar fashion, the adaptation of a highly proliferative, anti-apoptotic pathological state in activated fibroblasts has been extensively described in organ fibrosis [70,71,72]. Furthermore, our study shows for the first time that targeted p53-MDM2 interaction inhibition therapy can reverse dysfunctional proliferation, apoptosis, and fibrosis in glaucomatous LC cells. Importantly, our study suggests targeting the p53-MDM2-ubiquitin-proteasomal system in glaucomatous lamina cribrosa fibrosis may lead to future novel therapeutic interventions.

## 4. Materials and Methods

### 4.1. LC Cell Culture and Characterization

Primary human lamina cribrosa cells were received as a generous gift from Novartis^®^ (Cambridge, MA, USA). Freshly thawed primary LC cells were utilized from age-matched normal control healthy donor eyes (NLC) (no history of glaucoma, ocular disease, or other neurological conditions) and donor eyes with confirmed primary open-angle glaucoma (GLC) (n = 3 donors per group). Anonymous donors from regional eye banks were acquired and utilized in accordance with the Declaration of Helsinki.

Human donor eyes were obtained within 24 h of death. The LC region of the ONH (approximately 1 mm) was dissected for tissue explant culture. LC cells were isolated from ONH astrocytes through the use of specific serum-rich cell culture media [73,74]. LC cells were characterized through positive expression of elastin, fibronectin, laminin, and collagen types I, III, and IV [75,76] and the hallmark fibroblast marker αSMA [77]. LC cells showed negative expression of astrocyte marker GFAP and microglial marker ionized Ca^2+^ binding adapter molecule-1 (IBa-1) [78]. Cultured LC cells were maintained at 37 °C in 95% humified air with 5% CO_2_ in low-glucose DMEM (D5746, Sigma-Aldrich, St. Louis, MO, USA). supplemented with 10% (*w*/*v*) heat-inactivated FBS (F9665, Sigma Aldrich, Arklow, Ireland), 1% penicillin-streptomycin (10,000 units penicillin/mL and 10 mg streptomycin/mL) (P4333, Sigma-Aldrich, St. Louis, MO, USA), and 1% (4 mM) L-glutamine (G7513, Sigma-Aldrich, St. Louis, MO, USA). Cell media was replaced every 48–72 h until LC cells reached 90% confluency when they were passaged as needed. LC cells used in experimental procedures were between passages 3–8.

### 4.2. LC Cell Treatment

To determine the treatment effect of p53-MDM2 interaction inhibitor RG-7112 (Nutlin 3a), NLC and GLC cells (n = 3 donors per group) were incubated for 48 h in standard cell culture conditions (37 °C humidified, 5% CO_2_ atmosphere) with serum-complete media. This equated to approximately 90% cell confluency; serum-complete media was then removed, and cells were incubated overnight in a serum-free medium to ensure equivocal cell cycle arrest prior to treatment. RG-7112 (ab235500, Abcam, Cambridge, UK) was reconstituted in dimethyl sulfoxide (DMSO) (D8418, Sigma-Aldrich, St. Louis, MO, USA), and LC cells were treated at 10 μmol/L (μM) for 48 h.

The concentration of RG-7112 used in human LC cells (normal and glaucoma) cellular treatments was guided through a literature review [19,27,45,46] and formally evaluated through the utilization of a trypan blue exclusion test of cell viability [44]. Through the use of 0.4% trypan blue (SV30084.01, Cytiva HyClone^TM^, Thermo Scientific^TM^, Waltham, MA, USA) and a hemocytometer (Z359629, Bright-Line™ hemocytometer, Sigma-Aldrich, St. Louis, MO, USA), we were able to assess the percentage of viable cells (%) and the dose-dependent cytotoxic effect of RG-7112 in NLC and GLC cells (n = 3 donors per group) that received either no treatment (control) or varying concentrations of RG-7112 treatment (10 μM (μmol/L), 50 μM, 100 μM, 200 μM, and 500 μM).

### 4.3. Real-Time Quantitative Polymerase Chain Reaction

Ribonucleic acid (RNA) was isolated from treated/non-treated NLC and GLC cells using extraction agent TRIzol (93289, TRI Reagent^®^ Solution, Sigma-Aldrich, St. Louis, MO, USA),phase separation with chloroform, and precipitation by isopropanol. RNA (RNA) (4 μL) was reverse transcribed to complementary deoxyribonucleic acid (cDNA) using enhanced avian reverse transcriptase (AMV-RT) (1 μL) with corresponding 10X reaction buffer for AMV-RT (2 μL) (A4464 Sigma-Aldrich, St. Louis, MO, USA), oligo deoxythymine (Oligo(dT)23) (1 μL) (O4387, Sigma-Aldrich, St. Louis, MO, USA), deoxyribonucleotide triphosphates (dNTPs) (1 μL) (D7295, Sigma-Aldrich, St. Louis, MO, USA) and RNase-free H_2_O (11 μL). This process was performed in a GeneAmp^®^ PCR System 2400 Thermocycler (PerkinElmer, Shelton, CT, USA).

Real-time qualitative polymerase chain reaction (RT-qPCR) was performed to measure gene transcription levels in LC cells using LightCycler^®^ 480 Instrument II and accompanying software (Roche, Basel, Switzerland). Individual wells on a 96-well microplate (Roche, Basel, Switzerland) consisted of 5 μL LightCycler^®^ 480 SYBR Green I Master Mix (04707516001, (Roche, Basel, Switzerland), 3 μL RNase-free H_2_O, 0.5 μL forward primer, 0.5 μL reverse primer, and 1.5 μL cDNA. Negative controls and H_2_O-only wells were utilized. Standard qPCR cycling conditions were performed using the following steps: (1) denaturation at 95 °C for 5 min. (2) 45 cycles of amplification, with each cycle comprising denaturation at 95 °C for 10 s, annealing at 60 °C for 20 s, and elongation at 72 °C for 20 s. (3) Determination of melting curve by 95 °C for 5 s, 65 °C for 1 min, then continuous heating to 97 °C with 5 acquisitions/°C. (4) Finally cooling for 30 s at 40 °C.

All gene expression levels were expressed as the difference in cycle threshold (Ct) values (ΔCt) normalized to reference gene 18S according to the method of Livak [79]. Original Ct values above 40 cycles were deemed to be unreliable and designated as no detectable expression. RT-qPCR data are presented as the mean gene expression ± standard deviation (SD) for NLC and GLC (n = 3 donors per group). Gene expression levels for RG-7112-treated/non-treated NLC and GLC groups (n = 3 donors per group) are displayed in a similar fashion. Each experiment was performed in triplicate for experimental reliability (N = 3). The sequences of specific PCR primers (100 nM), including reference gene 18S, are listed in Table 1.

### 4.4. Western Blotting

To quantify protein expression levels of p53 and MDM2 in RG-7112-treated/non-treated NLC and GLC cells, western blotting was performed. When LC cells reached 90% confluency in T-75 cm^2^ tissue culture flasks, they were washed twice with 10 mL of ice-cold Dulbecco’s phosphate buffered saline (PBS) (D8537, Sigma-Aldrich, St. Louis, MO, USA) and then scraped into 1 mL ice-cold PBS using a chilled plastic cell scraper. This was followed by centrifugation at 4 °C (14,000 rpm (800× to 1000× *g*) for 10 min) to create a cell pellet. PBS supernatant was then removed, and the cell pellet was then resuspended and incubated for 5 min in 50 μL ice-cold modified radioimmunoprecipitation assay (RIPA) buffer (R0278, Sigma-Aldrich, St. Louis, MO, USA) supplemented with protease inhibitor cocktail (PIC) (P8340, Sigma-Aldrich, St. Louis, MO, USA) and phosphatase inhibitor cocktail (PhIC) (PS726, Sigma-Aldrich, St. Louis, MO, USA). Cell Iysates were then clarified by centrifugation at 4 °C (14,000 rpm for 10 min). The protein-containing supernatant was collected, flash-frozen, and stored at −80 °C prior to experimental analysis.

Cell lysate total protein concentration was determined using a Bradford assay (B6916, Sigma-Aldrich, St. Louis, MO, USA) with bovine serum albumin (23210, Pierce^TM^, Thermo Scientific^TM^, Waltham, MA, USA) utilized as the standard [80]. Prior to sodium dodecyl sulfate-polyacrylamide gel electrophoresis (SDS-PAGE), equal quantities of protein sample (15 μg/lane) were combined with Laemmli’s sample buffer (S3401, Sigma-Aldrich, St. Louis, MO, USA) and boiled at 95 °C [81]. 10 µL of molecular weight ladder (94964, BLUeye Prestained Protein Ladder, Sigma-Aldrich, St. Louis, MO, USA) was added to the first well, followed by cell samples to the remaining wells. SDS-PAGE was run at 100 V for 90 min using 8, 10, or 12% SDS-polyacrylamide separating gels in running buffer, followed by protein transferred to 100% methanol-reinforced nitrocellulose membranes (420101, Duralon-UV™ membranes, StrateGene^®^, La Jolla, CA, USA) in transfer buffer at 100 volts for 45 min at 4 °C. To prevent non-specific antibody binding, membranes were blocked using 3% (*w*/*v*) Marvel (non-fat milk) in tris-buffered saline-tween (TBST) at room temperature for 60 min. To enable protein detection, membranes were then incubated overnight at 4 °C in primary antibody diluted in 3% (*w*/*v*) Marvel/TBST with gentle rocking. Table 2 outlines the primary/secondary antibodies and their concentrations used in this work.

Following overnight incubation, the primary antibody was removed, and each membrane was washed three times in TBST. Membranes were then incubated with an IgG-horseradish peroxidase (HRP)-conjugated secondary antibody (1:2000 with 5% (*w*/*v*) Marvel/TBST) for 60 min at room temperature. HRP-conjugated secondary antibodies used were appropriate to the corresponding host species used for primary antibodies. Lastly, secondary antibodies were removed, and membranes were washed again three times in TBST and a final wash with TBS.

To enable chemiluminescent detection, membranes were developed through 2 min of incubation with enhanced chemiluminescence (ECL) reagents (10005943, Pierce^TM^, Thermo Scientific^TM^, Waltham, MA, USA). Chemiluminescent detection was performed using an electronic chemiluminescence imaging system (c-DiGiT Blot Scanner, LI-COR Biosciences, Lincoln, NE, USA) with accompanying software (Image Studio^TM^ 6.0 Software). This process was repeated with membranes re-probed with an anti-β-actin primary antibody as a loading control. Band size and density were quantified, relative to β-actin levels, through densitometry analysis performed using ImageJ software (Version 2, National Institutes of Health, Bethesda, MD, USA).

### 4.5. Cell Viability Assay

To assess cell viability and the cytotoxicity of RG-7112, we utilized a MTT tetrazolium reduction assay (3-(4,5-dimethylthiazol-2-yl)-2,5-diphenyltetrazolium bromide) (CT02, Millipore, Burlington, MA, USA). In preparation, 10 mL of PBS (pH 7.4) was added to MTT and stored overnight in the dark at 4 °C to allow for crystals to fully dissolve.

Treated/non-treated NLC and GLC cells (1 × 10^4^ cells/well) (n = 3 donors per group) were seeded in triplicate into 96-well plates. After 48 h of incubation in a 37 °C humidified, 5% CO_2_ atmosphere, serum-complete media was removed, and cells were incubated overnight in serum-free medium. Cells were then rinsed with sterile PBS and were assigned into untreated (control) or treated wells. Treatment wells received 10 μmol/L (μM) RG-7112 for 48 h in 100 μL 10% FBS serum-rich medium, with control wells receiving 100 μL 10% FBS serum-rich medium only.

Following 48 h, 10 μL of MTT (with PBS as described above) was added to each well using a multi-channel pipettor. Gentle tapping on the side of the tray ensured mixing. Approximately 96-well plates were again incubated at 37 °C for 4 h to allow mitochondrial dehydrogenase to cleave/convert MTT to insoluble formazan crystals. Furthermore, negative control wells were utilized, containing 100 μL of serum-complete media (cell-free) that also received 10 μL of MTT. Following this, 100 μL of isopropanol with 0.04 N HCl was added to each well using a multichannel pipettor, with repeated pipetting ensuring adequate mixing.

Absorbance was then read using a SpectraMax^®^ Plus 384 microplate spectrophotometer and accompanying software, SoftMax^®^ Pro 6.2.2 (Molecular Devices, San Jose, CA, USA), at a test wavelength of 570 nm and a reference wavelength of 630 nm. To enable normalization of results, reference wavelengths and control values for each group were subtracted from the test wavelength absorbance result. As per the manufacturer’s instructions, a single absorbance reading following 4 h of MTT incubation was captured. Each experiment was performed in triplicate (n = 3 donors per group) and repeated three times to ensure reproducibility of results (N = 3).

### 4.6. Cell Proliferation Assay

To assess cellular proliferation rates in NLC and GLC cells at baseline and with RG-7112 treatment, we used the CellTiter 96^®^ AQueous One Solution Cell Proliferation Assay (G3582, Promega Corporation, Madison, WI, USA) as per the manufacturers’ instructions. This assay utilizes a tetrazolium compound methyl thiazolyl tetrazolium salt (MTS) [3-(4,5-dimethylthiazol-2-yl)-5-(3-carboxymethoxyphenyl)-2-(4-sulfophenyl)-2H-tetrazolium] with an electron coupling reagent PES (phenazine ethosulfate).

Cells from treated/non-treated NLC and GLC cell groups were seeded in triplicate in a 96-well culture microplate (1 × 10^4^ cells/well) (n = 3 donors per group). Each well received 100 μL of serum-complete culture media for 48 h, then cells were serum-starved overnight. Cells were kept at 37 °C in a humidified 5% CO_2_ cell culture incubator. Cells were then rinsed with sterile PBS and were assigned into untreated (control) or treated wells. Treatment wells received 10 μmol/L (μM) RG-7112 for 48 h in 100 μL 10% FBS serum-rich medium, with control wells receiving 100 μL 10% FBS serum-rich medium only.

When evaluating baseline cellular proliferation rates in untreated NLC/GLC cells, cells were assessed at 0, 72, 96, and 120 h of total incubation. When evaluating RG-7112 treatment, cells were assessed at 96 h total incubation time (48 h of treatment). At each desired timepoint, 20 μL of CellTiter 96^®^ was added to the relevant wells of the 96-well assay plate containing the samples with a multi-channel pipettor. The plate was then incubated at 37 °C for 1 h in a humidified, 5% CO_2_ atmosphere. The plates were gently shaken to ensure complete solubilization of MTS, and cells were permitted to settle down for 10 min at room temperature. Furthermore, negative control wells were utilized containing 100 μL of serum complete media (cell-free) that also received 20 μL of CellTiter 96^®^.

Absorbance was then read using a SpectraMax^®^ Plus 384 microplate spectrophotometer, and accompanying software, SoftMax^®^ Pro 6.2.2 (Molecular Devices, USA), was used to read and record the absorbance at a wavelength of 490 nm. This experiment did not require a reference wavelength. As per the manufacturer’s instructions, a single absorbance reading following 1 h of MTS incubation was captured. Each experiment was performed in triplicate (n = 3 donors per group) and repeated three times to ensure reproducibility of results (N = 3). To enable normalization of results, the absorbance values for control wells for each group were subtracted from the test wavelength result.

### 4.7. Immunofluorescence

Immunofluorescence microscopy was performed on NLC and GLC cells to analyze if cellular localization varied (cytoplasmic vs. nuclear) in the different cell groups. NLC and GLC cells (n = 3 donors per group) were seeded into the Nunc Lab-Tek II Chamber Slide System (Thermo Scientific, Dublin, Ireland) and incubated in standard cell culture conditions in serum-complete culture media for 24 h, then serum-starved overnight. Cells were then fixed in 100% ice-cold methanol for 5 min and blocked for 1 h at room temperature in sterile PBS with 0.1% Triton X-100 and 5% normal goat serum (Thermo Scientific, Dublin, Ireland). Samples were subsequently probed for p53 (ab-26, 1:1000 dilution in blocking buffer) and MDM2 (sc-965, 1:50 dilution in blocking buffer) overnight at 4 °C, then washed three times with PBS and incubated for 2 h at room temperature with a species-specific secondary antibody (A10521, 1:500 dilution in PBS) (Thermo Scientific, Dublin, Ireland). Following further PBS washes, samples were washed with 4ʹ,6-diamidino-2-phenylindole (DAPI) (1:5000 dilution) to enable nuclear localization. Samples were then mounted to slides with aqua-polymount (Polysciences, Warrington, PA, USA). Images were then captured using a confocal microscope (Zeiss LSM 710; Zeiss, Oberkochen, Germany) with associated imaging software (ZEN Lite).

Images were quantified using ImageJ. First, images were imported, and regions of interest were drawn around the nuclei and cell membrane for each of the ten cells for both NLC and GLC monolayers. By determining the exclusive pixels for the regions of interest, the integrated density and area were then calculated separately for the cytoplasmic and nuclear compartments. The corrected total cell fluorescence (CTCF) was then determined for each compartment and plotted.

### 4.8. Statistical Analysis

All experimental procedures were performed in triplicate (technical replicates, N = 3) from three independent eye donors per group (NLC and GLC) (biological replicates, n = 3). Data analysis was performed using IBM SPSS Statistics Version 24 and is presented as mean ± standard deviation. The unpaired two-tailed *t*-test was used to analyze data from two independent groups (e.g., NLC and GLC groups). The paired two-tailed *t*-test was used to analyze data from correlating samples (e.g., proliferation assay at 0, 72, 96, and 120 h). A two-way ANOVA with Sidak’s multiple comparisons was applied to the data with more than one factor (e.g., NLC vs. GLC; RG-7112 vs. vehicle). Statistical significance was set at *p* < 0.05 and is denoted by * *p* < 0.05, ** *p* < 0.01, and *** *p* < 0.001 in graphical representation. “n” indicates the number of eye donors, which was set at 3 independent donors per group.

## Figures and Tables

**Table 1:** List of genes targeted and F and R primer sequences (Sigma Aldrich, Arklow, Ireland).

Target Gene		Sequence (5’-3’)
*p53*	F	GCCCAACAACACCAGCTCCT
	R	CCTGGGCATCCTTGAGTTCC
*MDM2*	F	GCAGTGAATCTACAGGGACGC
	R	ATCCTGATCCAACCAATCACC
*COL1A1*	F	GATGTGCCACTCTGACTGG
	R	GGGTTCTTGCTGATGTACCAG
*αSMA*	F	CTGTTCCAGCCATCCTTCAT
	R	CCGTGATCTCCTTCTGCATT
*BAX*	F	GGTTGTCGCCCTTTTCTA
	R	CGGAGGAAGTCCAATGTC
*BCL-2*	F	GATGTGATGCCTCTGCGAAG
	R	CATGCTGATGTCTCTGGAATCT
*Fas*	F	TGAAGGACATGGCTTAGAAGTG
	R	GGTGCAAGGGTCACAGTGTT
*Caspase-3*	F	AATTGCCTCCACACCTTCAC
	R	TCACCAAGCTGCTCATCAAC
*18S*	F	GTAACCCGTTGAACCCCATT
	R	CCATCCAATCGGTAGTAGCC

**Table 2:** Primary and secondary antibodies used for western blotting.

Target Protein	Host Species	Target Species	Blocking Reagent	Concentration	Product Code	Secondary Antibody
β-actin	Mouse	Human	3% milk	1:1000	Ab8226 (Abcam, Cambridge, UK)	Anti-mouse sc-2005 (Santa Cruz Biotechnology, Santa Cruz, CA, USA)
p53	Mouse	Human	3% milk	1:1000	Ab26 (Abcam, Cambridge, UK)	Anti-mouse sc-2005 (Santa Cruz Biotechnology, Santa Cruz, CA, USA)
MDM2	Rabbit	Human	3% milk	1:1000	Ab260074 (Abcam, Cambridge, UK)	Anti-rabbit ab205718 (Abcam, Cambridge, UK)
Caspase-3	Mouse	Human	3% milk	1:1000	Sc-7272 (Santa Cruz Biotechnology, Santa Cruz, CA, USA)	Anti-mouse sc-2005 (Santa Cruz Biotechnology, Santa Cruz, CA, USA)
αSMA	Mouse	Human	3% milk	1:1000	Sc-53015 (Santa Cruz Biotechnology, Santa Cruz, CA, USA)	Anti-mouse sc-2005 (Santa Cruz Biotechnology, Santa Cruz, CA, USA)

## Data Availability

Data are available upon reasonable request.
